# A Comparative Study of Sheep Breeds: Fattening Performance, Carcass Characteristics, Meat Chemical Composition and Quality Attributes

**DOI:** 10.3389/fvets.2021.647192

**Published:** 2021-03-19

**Authors:** G. M. Suliman, A. N. Al-Owaimer, A. M. El-Waziry, E. O. S. Hussein, K. Abuelfatah, A. A. Swelum

**Affiliations:** ^1^Department of Animal Production, College of Food and Agriculture Sciences, King Saud University, Riyadh, Saudi Arabia; ^2^Department of Meat Production, Faculty of Animal Production, University of Khartoum, Khartoum North, Sudan; ^3^Department of Animal Production and Fishery, Faculty of Agriculture, University of Alexandria, Alexandria, Egypt; ^4^Department of Theriogenology, Faculty of Veterinary Medicine, Zagazig University, Zagazig, Egypt

**Keywords:** sheep, breed, carcass, meat quality, muscle

## Abstract

Fattening performance, Carcass characteristics, chemical composition, and meat quality were evaluated in three sheep breeds: Awassi, Harri, and Najdi. Forty-five lambs of similar weight and age were raised for 90 days under similar conditions. The Harri and Najdi breeds had higher dressing-out percentages than Awassi sheep. The Awassi and Harri breeds had thicker backfat than the Najdi breed. No significant difference was found in moisture, protein, and intramuscular fat among the breeds. However, the Harri breed had a higher ash content than the Awassi and Najdi breeds. The Najdi breed had higher ultimate pH and lower cooking loss than the Awassi and Harri breeds. Awassi and Harri sheep had a higher myofibril fragmentation index, longer sarcomere length, and lower hardness and chewiness than Najdi sheep. Subjectively, no significant differences were detected between the breeds, except for flavor intensity while the Awassi sheep were rated in between and not significantly different. In conclusion, breed affected carcass characteristics, meat composition, and the quality of sheep. The dressing yield was higher in Harri and Najdi than Awassi sheep. Awassi sheep showed superior meat quality characteristics followed by Harri sheep. However, Najdi sheep had the best cooking loss percentage and flavor intensity score.

## Introduction

The primary livestock species producing red meat in Saudi Arabia are sheep, goats, cattle, and camels. Their total population is estimated to be 13,444,435 heads (1), distributed as follows: 9,055,438, 3,563,017, 354,276, and 471,704 heads, respectively. Therefore, sheep represent the majority (72%) of the livestock population, although the Kingdom of Saudi Arabia imports large numbers of sheep to satisfy its needs. Notably, sheep meat tops the preferred meat list of Saudi citizens followed by camel meat. Saudi Arabia don't produce sheep meat enough, a part is imported and Australia is the main source of this importation. The Business Monitor International (BMI) (2) reported that there was an expanding market for Australian red meat exports to Saud Arabia in the first 5 months of 2013, with estimated shipments up 171% annually. In this context, mutton exports have grown to a total of 7,584 tones, projecting an increase of 65% for the same period. The only way to shorten the distance between production and consumption, aiming at lowering the importation rates of sheep meat is to prize our assets, following the economic principle. The first step to follow is evaluating sheep breeds for their performance, productivity, the differences between them, the points of strength and weakness, and the quality of their products. Then, based on the outcomes, strategic plans and short- and long-term visions can be developed. It is hypothesized that the carcass characteristics, meat chemical composition and quality attributes can be varied depend on sheep breed. Moreover, the information available about meat quality characteristics of Awassi, Harri, and Najdi sheep is scant, though these are the main sheep breeds in Saudi Arabia being adaptive to local environment, resistant to indigenous parasites and diseases, have good meat producing ability and much preferred by the natives compared to the other sheep breeds. Surprisingly enough, there is no study (up-to-date) comparing theses three sheep breeds under search regarding their fattening performance, carcass characteristics and meat quality; while, some of them gained higher standing among consumers and in markets than the others without any solid base and facts. Hence, the necessity arises to compare between these breeds to explore their exact capabilities and particularities. Therefore, this study was conducted to evaluate carcass characteristics, meat chemical composition and, quality attributes of Awassi, Harri, and Najdi sheep breeds.

## Materials and Methods

### Animals, Housing, Feed, and Feeding

The experiment was conducted at the farm of the Department of Animal Production, College of Food and Agricultural Sciences, King Saud University, Riyadh, Saudi Arabia (24.8051° N, 46.5203° E). Three indigenous Saudi sheep breeds, Awassi (locally known as Ne'aimi), Harri, and Najdi, were used in this comparative study. A total of 45 intact lamb males (15 animals of each breed), with an age ranged from 84 to 95 days old and weight ranged from 23.40 to 25.87 kg, were included in this trial. The lambs were ear-tagged, treated against internal and external parasites, and housed in partially shaded pens supplied with individual feeding and watering facilities and subjected to a feeding period of 90 days after an adaptation period of 14 days. All the lambs were kept under the same conditions and fed the same diet, which was formulated to meet the nutrient requirements of lambs (3) and was offered *ad libitum*. The animals were fed iso-nitrogenous and iso-caloric commercial pelleted feed. The feed ingredients included alfalfa hay, maize, barely, soybean meal, minerals + trace elements supplements and vitamins. The feed chemical composition was 13.75, 8.16, 2.11, 8.6, and 67.38% for crude protein (CP), crude fiber (CF), ether extract (EE), ash and nitrogen free extract (NFE), respectively. Metabolizable energy (ME) of feed was 2.7 Mcal/kg. Drinking water and salt licks were made available around the clock. The experiment was conducted following the guidelines outlined by the Ethical Committee (Ethics Reference No. KSU-SE-20-17).

### Fattening Performance

Live weight was recorded at the start and end of the experimental period (90 days). Feed intake was determined daily as the difference between the amounts of feed offered and refusals. Average daily intake (ADI), average daily gain (ADG), and feed conversion ratio (FCR) were calculated.

### Animal Slaughtering, Carcass Evaluation, and Muscle Sampling

At the end of fattening period (90 days), eight animals were randomly selected from each breed and slaughtered following the approved Halal meat protocol directed by the legislation of Islam. Carcass and non-carcass components were weighed immediately after slaughter, and the weight of the digestive contents was computed as the difference between the full and empty digestive tract. The empty body weight (EBW) was computed as the difference between the slaughter weight and weight of digested content. All carcasses were chilled (at 4°C) for 24 h. Then, the cold weight was taken, and the carcasses were split into two halves from the pelvis to the neck along the vertebral column. The left side of the chilled carcass was cut between the 12th and 13th ribs to determine the extent of the rib-eye area and thickness of the back and body wall fats. The *Longissimus thoracis* (LT) muscles from the 9th to 12th thoracic vertebrae of both sides were removed for analyses. In brief, reading of pH and color components were performed on the steak. Drip loss and water-holding capacity tests needed meat samples of around 20 and 2 g, respectively. The cooking loss test needed a steak of approximately 2.5 cm (around 300 g). Texture profile analysis needed a sample similar to that of CL test (300 g). The shearing force was performed using the same sample of CL determination after being cooked. Sarcomere length and MFI tests required 10 and 4 g, respectively. Meat chemical composition and taste panel tests needed meat samples of around 100 and 200 g, respectively.

### Meat Chemical Composition

The LT muscle was used to estimate the moisture, crude protein, crude fat, and ash based on the protocol outlined by GASTAT (1).

### Meat Quality Analysis

#### pH and Color Measurements

The ultimate pH (pHu) of each carcass was measured at 24 h postmortem using a portable pH meter (Model pH 211, Hanna Instruments, Woonsocket, Rhode Island, USA) consistently on the left *Longissimus* muscle caudal to the 12th rib. LT samples were analyzed for color characteristics: lightness (L^*^), redness or red–green scale (a^*^), and yellowness or yellow–blue scale (b^*^). The color measurements were assessed using a colorimeter (Konica Minolta, CR-400-Japan; Measuring aperture: 8 mm; Illuminant: CIE D65; Observer angle: CIE 2° Standard Observer). Before measuring, a blooming time of 30 min was applied. Three readings were taken on the muscle surface, and a mean value was processed. Values of a^*^ and b^*^ were used to calculate color saturation (chroma), hue angle (H°) and b^*^ to a^*^ ratio based on the following equations: chroma (C^*^) = (a^*2^ + b^*2^)^1/2^ and hue angle (H°) = tan^−1^ (b^*^/a^*^) described by Mancini and Hunt (4) and Olfaz et al. (5).

#### Cooking Loss

Cooking loss (CL) was calculated following the procedures described by Al-Owaimer et al. (6). The samples were placed in an electric commercial stainless-steel grilling oven and cooked at 200°C to an internal temperature of 70°C. After cooking, the steaks were cooled down to room temperature (20°C), surface dried with filter paper, reweighed, and the CL was expressed as the percentage weight change.

#### Water-Holding Capacity

The water-holding capacity (WHC) was determined following the methodology described by Wilhelm et al. (7). A meat sample of approximately 2 g was analyzed in duplicate. Initially, the sample was placed between two filter papers and then left under a 10 kg weight for 5 min. Finally, the WHC was determined as the difference between the initial and final weight of the sample and expressed as a ratio relative to the original weight.

#### Drip Loss

To evaluate drip loss (DL), a meat sample of around 20 g was taken and placed in sealed polyethylene plastic bag, thereafter stored in a chiller at (4°C) for 24 h. Then, the sample was removed from the bag, gently wiped, and reweighed. The DL was expressed as a percentage of the weight change.

#### Myofibril Fragmentation Index

The myofibril fragmentation index (MFI) of the LT samples from the three breeds was calculated following Culler et al. (8). In brief, 4 g of the muscle sample was minced using scissors. Then, it was homogenized in a mixer with 40 ml of cold (2°C) MFI buffer. Thereafter, several washes were performed, and then, the absorbance of the resultant 0.5 mg/ml solution was read at 540 nm using a spectrophotometer (HACH DR/3000 Spectrophotometer, USA). The MFI of each sample was calculated by multiplying the absorbance at 540 nm by 200.

#### Sarcomere Length (SL)

The sarcomere length (SL) was performed following the method described by Cross et al. (9). Briefly, three longitudinal muscle samples (3 cm × 3 cm × 2 cm) were removed and stored in 5% glutaradehyde solution for 4 h at 4°C. The SL was then determined by laser diffraction.

#### Shear Force and Texture Profile Analysis

A 2.5-cm-thick muscle sample (approximately 300 g) was taken to perform the test. The sample was placed in an electric commercial stainless-steel grilling oven and cooked at 200°C to an internal temperature of 70°C. The internal temperature was adjusted by inserting a thermocouple probe (Ecoscan Temp JKT, Eutech Instruments, Pte Ltd., Keppel Bay, HarbourFront, Singapore) into the center of each steak. The shear force (SF) of the LT was assessed following Wheeler et al. (10). Three round cores (1.27 cm in diameter) were removed from each cooked muscle sample parallel to the longitudinal orientation of the muscle fibers. The SF was obtained as the maximum force (N/cm^2^) perpendicular to the fibers using a TA.HD texture analyzer (Stable Micro Systems, Surrey, UK) outfitted with a Warner–Bratzler attachment. The texture profile analysis (TPA) was conducted using the texture analyzer (TA.HD, Stable Micro Systems, Surrey, UK) fitted with a compression-plate attachment. Each sample underwent two cycles of 80% compression. The components determined were hardness, cohesiveness, springiness, and chewiness.

#### Sensory Evaluation

The test was performed using a 2.5-cm-thick meat steak (about 400 g). The meat samples were prepared and cooked under precise and uniform conditions, and then presented to panel members in specialized testing room. The taste panel room equipped with individual booths and prepared to meet all the specifications required to perform accurate and reliable sensory evaluation test as room temperature and ventilation, booth dimensions, red color light to mask color differences between meat samples, and other requirements. The room was connected to a small kitchen for sample preparation and handling. The kitchen was equipped with electrical grilling oven, warming food cabinets, refrigerator, and general kitchen supplies. The meat sample was placed in an electric commercial stainless-steel grilling oven and cooked at 200°C to an internal temperature of 70°C. The internal temperature of the sample was monitored by inserting a thermocouple probe (Ecoscan Temp JKT, Eutech Instruments, Pte Ltd., Keppel Bay, HarbourFront, Singapore) into the center of each steak. The category scaling method was used to categorize the meat samples on an 8-point category scale following Suliman et al. (11, 12). A panel of eight trained panelists assessed the cooked meat samples for flavor, tenderness and juiciness. The panel members were chosen for their ability to distinguish meat attributes under consideration and trained in how to score different characteristics. Each panelist was asked to score four samples per treatment at each session. Two sessions were held to complete the test. The samples were about 2 cm in size (~50 g) that presented in a disposable plastic container. The panelists were requested to avoid food and smoking 2 h before meat tasting. Water and crackers were available to remove any residual flavor of the previous samples.

### Statistical Analysis

Differences in the means of the different treatment groups were tested using analysis of variance in SPSS® software program version 21 (SPSS, Chicago, IL), while separation of the means was performed using Duncan's Multiple Range Test. Data were expressed as the mean ± standard error of the mean (SEM).

## Results and Discussion

### Fattening Performance

All the experimental animals started the growth period that extended for 90 days with an initial live weight (ILW) of approximately 24.56 kg. The ILW did not differ significantly between the treatment animals. On the other hand, the final live weight (FLW) differed between the treatment groups where Awassi breed attained the highest (*P* < 0.05) FLW followed by Najdi then Harri. Average daily intake (ADI), average daily gain (ADG) and feed conversion ratio (FCR) were significantly different between the sheep breeds. The Awassi sheep breed reported the highest (*P* < 0.05) ADI followed by Najdi > Harri, and the best (*P* < 0.05) ADG and FCR compared to the other two sheep breeds (Table 1). The superior FLW of Awassi sheep is attributed to the eminent ADG and FCR over Najdi and Harri breeds. The Harri breed showed a growth performance that located intermediate between the other two breeds.

**Table 1:** Fattening performance of the three sheep breeds fed the experimental diet (*n* = 15/group).

**Item**	**Breed**			**Limits**		**SEM**
	**Awassi**	**Harri**	**Najdi**	**Minimum**	**Maximum**	
Initial live weight, kg	24.31	24.70	24.68	23.40	25.87	0.23
Final live weight, kg	50.52^a^	44.51^b^	48.54^a^	42.00	53.70	0.86
ADI kg/day	1.69^a^	1.41^b^	1.64^a^	1.35	1.87	16.12
ADG kg/day	0.31^a^	0.24^b^	0.28^a^	0.20	0.34	10.11
FCR	5.49^b^	6.91^a^	5.95^b^	5.16	6.81	0.20

*ADI, Average daily intake; ADG, Average daily gain; FCR, Feed conversion ratio*.

a,b *Means within rows not sharing the same letter (s) differ significantly (p < 0.05)*.

### Slaughter Weights and Carcass Characteristics

At the end of experimental period (90 days), the slaughter weight and carcass characteristics of three sheep breeds at there shown in Table 2. The slaughter body weight (SBW), EBW, and hot carcass weight (HCW) were significantly (*P* < 0.05) greater in Awassi and Najdi breeds than the Harri breed. The Najdi breed had a higher cold carcass weight (CCW) than the Harri sheep (*P* < 0.05). The dressing-out percentage (DP) based on SBW or EBW is one of the primary variables used to evaluate carcass characteristics, and it has considerable economic importance. In small ruminants, the DP based on SBW ranged from 36 to 60%, and it was affected by different factors (13). In this study, the SBW-based and EBW-based DP were in the range of 47.6–50.8 and 51.75–54.96, respectively. These results are similar to those previously reported (2, 3, 6, 14). The Harri and Najdi breeds had significantly (*P* < 0.05) higher DP (SBW) and DP (EBW) than Awassi sheep. No significant difference was detected in the gut fill of the different sheep breeds (Table 3). Therefore, the differences in DP can be attributed to the effect of the breed; besides effects of carcass weight, non-carcass components and internal body fat. Several studies have indicated that breed is one of the primary factors affecting DP (2, 3, 6–8, 14, 15). While Peña et al. (16) indicated that carcass weight had a significant influence on DP and other carcass quality traits.

**Table 2:** Slaughter weight and Carcass characteristics of three sheep breeds (*n* = 8/group).

**Component**	**Breed**			**Limits**		**SEM**
	**Awassi**	**Harri**	**Najdi**	**Minimum**	**Maximum**	
SBW (kg)	50.53^a^	45.19^b^	49.54^a^	43.05	57.20	0.74
EBW (kg)	46.48^a^	41.70^b^	45.46^a^	39.50	51.50	0.64
HCW (kg)	24.04^a^	22.93^b^	24.69^a^	21.15	27.94	0.30
CCW (kg)	23.35^a,b^	22.39^b^	24.06^a^	20.67	27.10	0.29
DP (SBW)	47.60^b^	50.80^a^	49.50^a^	45.30	53.00	0.47
DP (EBW)	51.75^b^	54.96^a^	54.38^a^	49.40	57.40	0.51
CS%	2.90^a^	2.39^b^	2.53^a,b^	1.89	3.49	0.09
Rib-eye area (cm^2^)	7.77	8.94	8.67	6.07	11.90	0.30
Back fat (mm)	2.58^a^	1.99^a^	1.29^b^	0.88	3.94	0.16
Body wall fat (mm)	3.99	3.53	4.35	2.01	6.40	0.22

a,b *Means within rows with different superscripts are significantly different (p < 0.05)*.

*SBW, slaughter body weight; EBW, empty body weight; DP (EBW), dressing percentage per empty body weight; DP (SBW), dressing percentage per full body weight; HCW, hot carcass weight; CCW, cold carcass weight; CS, chiller shrinkage*.

**Table 3:** Non-carcass components and fat content of the three sheep breeds (*n* = 8/group).

^*^Component%	**Breed**			**Limits**		**SEM**
	**Awassi**	**Harri**	**Najdi**	**Minimum**	**Maximum**	
Head	7.57	6.94	7.58	6.15	8.56	0.15
Heart	0.69	0.60	0.68	0.54	0.90	0.02
Lungs and trachea	2.30^a^	1.81^b^	2.18^a^	1.52	3.16	0.08
Liver	3.21	2.95	3.09	2.24	3.69	0.07
Spleen	0.29	0.27	0.29	0.20	0.38	0.01
Kidneys	0.51^a^	0.44^b^	0.48^a,b^	0.37	0.57	0.01
Tail	11.59	13.78	12.64	6.58	21.23	0.71
Stomach empty	7.97	7.05	7.18	5.59	8.90	0.22
Intestine empty	6.27^a^	4.43^c^	5.43^b^	3.53	7.84	0.21
Gut fill	16.85	15.16	16.48	10.90	21.80	0.66
Pericardial fat	0.45	0.49	0.48	0.32	0.56	0.02
Omental fat	3.06	3.42	2.90	1.41	5.13	0.20
Mesenteric fat	2.09	1.66	1.62	0.90	3.78	0.14
KKCF	2.37	3.06	1.86	0.19	6.61	0.14

* *Components were computed as a percentage of hot carcass weight*.

a,b,c *Means within rows with different superscripts are significantly different (p < 0.05)*.

*KKCF, kidney knob and channel fat*.

There were no significant differences in chiller shrinkage (CS), rib-eye area, and body wall fat among the different sheep breeds. The Awassi and Harri breeds exhibited significantly (*P* < 0.05) thicker back fat than the Najdi breed. CS or evaporative weight losses have previously been reported to account for 2% of the HCW during the initial 24 h of chilling beef, pork, and lamb (17). Body fat cover is the primary variable linked with CS (18). In this study, the CS was higher than that reported by Greer and Jones (17). This could be due to low body wall fat in sheep breeds, which ranged from 3.53 to 4.35 mm. Generally, tropical sheep breeds tend to deposit less subcutaneous fat than temperate sheep breeds (19).

### Non-carcass Components and Fat Content

There were no significant differences between breeds for the head, heart, liver, spleen, tail, stomach (empty), and internal fat deposits (kidney knob and channel fat [KKCF], pericardial, omental, and mesenteric) (Table 2). The Awassi and Najdi breeds had heavier lungs and trachea than the Harri breed. The kidneys were heavier (*P* < 0.05) in the Awassi than Harri breed. The weight of the empty intestine varied significantly between sheep breeds: the Awassi breed had the heavier intestine, followed by the Najdi breed.

### Meat Chemical Composition

The chemical compositions of the meat of the three sheep breeds are provided in Table 4. The moisture and protein contents of the meat ranged from 73.34 to 74.40% and 20.62 to 20.86%, respectively, with no significant differences between the three sheep breeds. The Awassi breed had the highest percentage of intramuscular fat (4.74%) followed by the Najdi breed (3.95%), and lastly, the Harri breed (3.84%). However, the difference was not significant. The ash content was significantly higher in the Harri breed (1.11%) than the Awassi and Najdi breeds, which had similar ash content (1.07%). In general, the chemical composition of meat is affected by numerous factors, including diet, carcass weight, and breed (10, 11, 13, 15, 17–27). The moisture content of the three sheep breeds was in the range reported by Abdullah and Qudsieh (28) for Awassi sheep. Our findings for protein content were in the range reported by Corazzin et al. (13) for sheep meat. In terms of the effect of sheep breed on protein content (29) did not find any effect of breed on meat protein. However, Rodrigues et al. (20) and Bjelanović et al. (30) found that breed has a significant effect on protein content. In the current study, the three sheep breeds had almost similar protein content, suggesting that breed has no effect on the protein content of sheep meat.

**Table 4:** Meat chemical composition of the three sheep breeds (*n* = 8/group).

**Composition %**	**Breed**			**Limits**		**SEM**
	**Awassi**	**Harri**	**Najdi**	**Minimum**	**Maximum**	
Moisture	73.34	74.40	74.37	70.73	75.85	0.28
Protein	20.86	20.64	20.62	19.48	21.97	0.12
Fat	4.74	3.84	3.95	1.28	8.29	0.35
Ash	1.07^b^	1.11^a^	1.07^b^	1.00	1.16	0.01

a,b *Means within rows with different superscripts are significantly different (p < 0.05)*.

The intramuscular fat content of sheep meat commonly ranges between 1.5 and 9.5% (31), depending on many factors. However, meat with a moderate quantity of intramuscular fat is preferred by the consumer (32). In the current study, the three sheep breeds had intramuscular fat ranging between 3.84 and 4.74%, which is considered a moderate preferred quantity of intramuscular fat (33). This result is inconsistent with the findings of (21, 34), who reported a higher intramuscular fat of Najdi than Awassi.

### Meat Quality Characteristics

Table 4 shows the meat quality characteristics of the three sheep breeds. The pHu values of the three sheep breeds ranged from 5.80 to 5.89. Generally, the pHu of the sheep meat declines from seven upon slaughter to reach approximately 5.3–5.8 at 24 h (35). In this study, there were significant differences between the breeds. The Najdi breed had a higher pHu than the Awassi (5.82) and Harri (5.80) breeds. Variation in pHu in different breeds was also reported by El Hassan et al. (22) and Hopkins and Fogarty (36), and this may be attributed to differences in glycogen levels in muscles and pre-slaughter stress (37).

The CL for the three sheep breeds ranged from 28.9 to 34.5%. Generally, the sheep meat had CL values ranging from 14 to 41% (13). The meat from Najdi sheep had a significantly lower CL (28.9%) than that of Harri (33.47%) and Awassi (34.54%) sheep. The effect of breed on the CL of sheep meat has also been reported by Mateo et al. (23) and Kuchtík et al. (29) and is probably because of the different pHu. In a living animal the muscle pH is approximately 7.2. After animal death, glycogen is broken down to lactic acid when muscle turns into meat. The ultimate pHu of meat, at 24 h post-mortem, can range from 5.2 to 5.8. Both the rate and extent of post-mortem pH fall will influence meat quality characteristics. A low ultimate pHu results in meat proteins having decreased water-holding capacity, while a higher ultimate pHu will give less cooking loss. In this study, the Najdi breed had the highest pHu and the lowest CL percentage. The WHC and drip loss of the three sheep breeds were in the range of 1.23–1.41 and 4.33–3.73, respectively, with no significant differences between breeds.

The color of meat is the most important quality attribute. The decision to purchase meat is affected more by the appearance of the meat than any other aspect of quality (4). There were no significant differences in the ultimate color characteristics of lightness (L^*^), redness (a^*^), and yellowness (b^*^) among the three sheep breeds. The values of a^*^ were in the range of 16.22 to 16.62, and the values of b^*^ were in the range of 4.91–5.76. These values were higher than those reported in the Omani sheep breed by Burke et al. (24) and Al-Khalasi and Mahgoub (38) and lower than the reported by Esenbuga et al. (39). The variation may be due to age and/or diet differences. The values of L^*^ and a^*^ color components reported in this study are looked acceptable as consumers consider fresh sheep meat with a^*^ and L^*^ values equal to or exceeding 9.5 and 34, respectively, as acceptable (40). Consumers consider fresh sheep meat with a^*^ and L^*^ values equal to or exceeding 9.5 and 34, respectively, as acceptable (40). The sheep breeds under investigation did not show any significant differences between them regarding chroma, b^*^/a^*^ ratio or hue angle. Awassi breed revealed the highest values of these components comparing to the other two breeds followed by Harri then Najdi. Once again, Harri located in-between the two breeds with an average color components' value. This may be attributed to the common ancestry of the three breeds. In a study (41) reported that Harri and Najdi breeds were categorized within the same gene pool based on a structure analysis. Moreover, a second study that evaluated the genetic diversity of these three breeds using microsatellite markers, concluded there was low population differentiation among the three sheep populations (42).

Tenderness is the most important eating quality characteristic, and it determines consumer acceptability (43). Meat tenderness depends on many intrinsic and extrinsic factors. Many studies have reported a positive correlation between meat tenderness and MFI (15, 17, 18, 44) and sarcomere length (45). Therefore, MFI and SL were investigated. As shown in Table 5, the different sheep breeds exhibited different MFI values, which ranged from 110.87 to 77.56. Awassi and Harri sheep had significantly higher MFI than Najdi sheep. The SL ranged from 1.63 to 1.85 μm with significant differences between the sheep breeds. The Awassi breed had the longest SL, and the Najdi breed had the shortest, while the Harri breed fell in between them. Sarcomere is the smallest contractile unit of a muscle fiber and serves as the basic force-producing machinery of striated muscles. The sarcomere length (SL) have only indirect effects on meat quality while an animal is living, but soon postmortem there will be pertinent impacts (46). The postmortal sarcomere length has marked effects on textural properties of raw and cooked meat, and on water-holding especially in raw meat as well as indirect effects on color and taste. This could explain the intermediate position of Harri breed between Awassi and Najdi breeds with respect to most investigated carcass and meat quality parameters in this study. The SL values observed in this study are similar to those reported by Gaili (19) and Devine et al. (47).

**Table 5:** Meat quality characteristics, myofibril fragmentation index (MFI) and texture profile analysis (TPA) of the three sheep breeds (*n* = 8/group).

**Characteristic**	**Breed**			**Limits**		**SEM**
	**Awassi**	**Harri**	**Najdi**	**Minimum**	**Maximum**	
*pH_*u*_*	5.82^b^	5.80^b^	5.89^a^	5.60	5.90	0.02
Cooking loss%	34.54^a^	33.47^a^	28.96^b^	22.12	41.81	0.86
WHC	1.23	1.38	1.41	0.23	1.53	0.05
Drip loss%	4.33	3.73	3.88	2.60	6.60	0.23
**Ultimate color values**						
L^*^	34.63	33.50	35.39	31.05	40.75	0.54
a^*^	16.62	16.49	16.22	14.24	18.98	1.40
b^*^	5.76	5.17	4.91	3.92	7.29	0.18
Chroma (C*)	17.60	17.29	16.97	14.87	20.08	0.30
b*/a* Ratio	0.35	0.31	0.30	0.22	0.40	0.01
Hue Angle (H°)	19.14	17.42	16.79	12.63	22.03	0.50
MFI	110.87^a^	104.0^a^	77.56^b^	45.70	153.55	6.27
SL (μm)	1.85^b^	1.79^a^	1.63^b^	1.26	2.10	0.04
^*^TPA						
Shear force (N/cm^2^)	32.17	29.03	28.34	18.54	38.93	0.13
Hardness (N/cm^2^)	3.83^b^	6.47^a^	6.67^a^	3.04	13.04	0.06
Springiness	0.58	0.60	0.61	0.49	0.70	0.01
Cohesiveness	0.53	0.50	0.53	0.41	0.59	0.01
Chewiness	0.13^b^	0.21^a,b^	0.23^a^	0.13	0.43	0.02

a,b *Means within rows with different superscripts are significantly different (p < 0.05)*.

* *TPA, texture profile analysis*.

The SF, which was defined as the maximum load needed to cut the meat perpendicular to fibers, is inversely associated with tenderness. Red meat is classified as tender until a SF of 34.72 N/cm^2^, intermediate from 40.01 to 52.96 N/cm^2^, and tough if the SF exceeds 57.86 N/cm^2^ (48, 49). In this study, the three sheep breeds had SF mean values ranging from 28.34 to 32.17 N/cm^2^. According to the above classification, the meat of three breeds is considered as tender meat. The Awassi breed had a particular quality with the highest MFI and SL values and lowest SF value. The three sheep breeds differed significantly (*P* < 0.05) in hardness and chewiness, but not in springiness and cohesiveness. Again, the Awassi breed showed the lowest value of hardness and chewiness followed by the Harri breed. The three sheep breeds differed significantly (*P* < 0.05) in hardness and chewiness, but not in springiness and cohesiveness. Again, the Awassi breed showed the lowest value of hardness and chewiness followed by the Harri breed. Nevertheless, it reported the highest (*P* > 0.05) shear force indicating less tender meat compared to the other two breeds. Hardness is the force needed to achieve a given deformation. It represents the hardness of the sample at first bite. While shear force is the force necessary to shear a piece of meat (50). Once again, Harri breed located intermediate between Awassi and Najdi breeds when texture profile parameters were considered.

### Sensory Evaluation

Sensory evaluation scores of the three sheep breeds are provided in Table 6. The sheep breeds only differed significantly (*P* < 0.05) in flavor intensity, and the Najdi breed gained the highest score followed by the Awassi breed.

**Table 6:** Sensory evaluation of the meat of three sheep breeds (*n* = 8/group).

Attribute^*^	**Breed**			**Limits**		**SEM**
	**Awassi**	**Harri**	**Najdi**	**Minimum**	**Maximum**	
Juiciness	5.23	4.88	5.36	4.00	6.40	0.13
Tenderness	5.58	5.46	5.85	4.50	6.80	0.13
Flavor intensity	5.53^a,b^	5.19^b^	5.89^a^	4.60	6.90	0.11

a,b *Means within rows with different superscripts are significantly different (p < 0.05)*.

* *Sensory evaluation was performed using an eight-point hedonic scale (1 = the least; 8 = the best)*.

## Conclusions

This study revealed that there were prominent differences between the sheep breeds under investigation. Awassi, Harri and Najdi sheep showed noticeable variations regarding carcass characteristics, meat composition and quality. Awassi breed reflected better meat quality attributes than the other two breeds, nevertheless, Harri appeared to have the best dressing yield. In addition, Harri has the less non-carcass components besides an intermediate body fat content that suits today's healthy eating behavior. It is a fact that Awassi breed has a good reputation among Saud citizens and being the favorable source of meat for a long time, but it is the time for Harri breed to gain his place. In conclusion, we recommend a future breeding program ending in a new cross-sheep breed that have the best of Awassi and Harri.

## Data Availability Statement

The raw data supporting the conclusions of this article will be made available by the authors, without undue reservation.

## Ethics Statement

The animal study was reviewed and approved by the King Saud University Ethical Committee (Ethics Reference No: KSU-SE-20-17).

## Author Contributions

GS and AE-W: conceptualization. AA-O and GS: methodology. EH and KA: software. AS and EH: investigation. KA and AE-W: data curation. GS and AS: writing—original draft preparation, review and editing. AA-O: visualization. All authors have read and agreed to the published version of the manuscript.

## Conflict of Interest

The authors declare that the research was conducted in the absence of any commercial or financial relationships that could be construed as a potential conflict of interest.
